# Autonomic Nervous System Dysregulation in Metabolic Syndrome: An Association With Hypertension and Cardiovascular Risk

**DOI:** 10.7759/cureus.98932

**Published:** 2025-12-10

**Authors:** Hoor Soomra, Asad Mukhtar, Fatima Asif, Ayesha Khalid, Sadia Noureen, Zeeshan Qamar, Usman Haider

**Affiliations:** 1 Cardiology, University Hospitals Dorset NHS Foundation Trust, Bournemouth, GBR; 2 Internal Medicine, Faisalabad Medical University, Faisalabad, PAK; 3 Internal Medicine, Shrewsbury and Telford NHS Trust, Telford, GBR; 4 Cardiology, Shaikh Zayed Hospital, Lahore, PAK; 5 Internal Medicine, Niazi Medical and Dental College, Sargodha, PAK; 6 General Internal Medicine, University Hospitals Bristol and Weston NHS Foundation Trust, Bristol, GBR; 7 Internal Medicine, Naas General Hospital, Naas, IRL

**Keywords:** autonomic nervous system, cardiovascular diseases, heart rate variability, hypertension, metabolic syndrome

## Abstract

Metabolic syndrome (MetS) is a cluster of cardiometabolic abnormalities, including abdominal obesity, insulin resistance, dyslipidemia, and elevated blood pressure, that increases the risk of type 2 diabetes and cardiovascular disease (CVD). Autonomic imbalance, characterized by increased sympathetic activity and reduced parasympathetic tone, is proposed to play an important role in the development of hypertension and adverse cardiovascular outcomes in individuals with MetS. This systematic review evaluates the association between autonomic nervous system (ANS) dysregulation and MetS. A systematic search was conducted in PubMed, Embase, Scopus, and Cochrane Library for studies published from January 2015 to September 2025. Eligible studies included human research that examined measures of autonomic function such as heart rate variability (HRV), baroreflex sensitivity, muscle sympathetic nerve activity, and plasma catecholamine levels at rest in individuals with MetS. Observational and interventional studies were included. Data were extracted and synthesized narratively. A total of 16 studies met the inclusion criteria. Most included studies reported reduced HRV, impaired baroreflex sensitivity, increased resting sympathetic nerve activity, and elevated plasma catecholamines in participants with MetS, suggesting a consistent association between ANS dysregulation and blood pressure elevation. However, causality could not be established due to the predominantly observational study designs. Current evidence indicates a significant association between autonomic dysfunction and MetS, particularly in relation to hypertension and increased cardiovascular risk. ANS biomarkers may support refined cardiometabolic risk stratification, although further prospective and mechanistic studies are needed to clarify causal pathways.

## Introduction and background

Metabolic syndrome (MetS) is a cluster of interrelated metabolic conditions characterized by central obesity, insulin resistance, dyslipidemia, and elevated blood pressure, defined according to standard criteria such as the International Diabetes Federation (IDF) and National Cholesterol Education Program Adult Treatment Panel III (NCEP ATP III) classifications [1]. MetS represents a major and growing public health challenge worldwide. Recent estimates suggest that MetS affects approximately 20-25% of the global adult population, with prevalence exceeding 30% in several Middle Eastern and South Asian regions where rapid urbanization and lifestyle transitions have intensified cardiometabolic risk [2, 3]. Moreover, hypertension, one of the core components of MetS, accounts for more than 10 million deaths annually, representing a leading contributor to cardiovascular morbidity and mortality [4].

Lifestyle determinants, including energy-dense diets, physical inactivity, and increasing urbanization, are strongly associated with the rising burden of MetS and hypertension. These factors accelerate visceral adiposity, impair insulin signaling, and promote systemic inflammation, contributing to the global expansion of MetS and its cardiovascular consequences [5]. MetS is therefore recognized as a complex disorder involving vascular, inflammatory, and neurohormonal processes that synergistically elevate the risk of type 2 diabetes mellitus (T2DM), myocardial infarction, stroke, and other cardiovascular events [6].

A growing body of evidence indicates that dysfunctional autonomic nervous system (ANS) regulation is a key mechanism linking MetS to the development and progression of hypertension. The ANS modulates involuntary physiological functions, including heart rate, vascular tone, and metabolic homeostasis, through its sympathetic and parasympathetic branches [7]. Sympathetic hyperactivation and reduced parasympathetic (vagal) tone have been consistently observed in MetS, leading to increased vascular resistance, endothelial dysfunction, and impaired blood pressure regulation [8]. These alterations are further amplified by adipose tissue-derived cytokines and free fatty acids, which stimulate central autonomic pathways and promote sympathetic overdrive, while diminished baroreceptor sensitivity reduces heart rate variability (HRV), a well-established marker of autonomic impairment [9, 10].

Autonomic imbalance not only contributes to the onset of hypertension but also heightens cardiovascular risk by promoting arterial stiffness, atherosclerotic progression, arrhythmogenesis, and reduced cardioprotective responses to stress [11]. Importantly, ANS dysfunction may emerge before clinically overt cardiovascular disease (CVD), suggesting its potential value as an early biomarker for risk stratification in individuals with MetS [12].

Despite increasing recognition of these associations, the precise mechanisms through which ANS dysregulation interacts with metabolic, vascular, and inflammatory pathways in MetS remain insufficiently synthesized in current literature. A clearer understanding of this relationship is essential for informing preventive and therapeutic strategies targeting autonomic function. Therefore, this review aims to examine the role of ANS dysregulation in MetS, with a particular focus on its contribution to the development of hypertension and subsequent cardiovascular risk. By clarifying these interconnected mechanisms, the review seeks to support future development of more targeted interventions for early risk reduction and improved cardiometabolic outcomes.

## Review

Methodology

Protocol and Reporting Standards

This systematic review followed the Preferred Reporting Items for Systematic Reviews and Meta-Analyses (PRISMA) 2020 statement [13]. The protocol was created prospectively, with preselected eligibility criteria, search terms, and appraisal strategies. While not registered formally on PROSPERO, procedures conformed to international best practices to maximize transparency and replicability. Decisions made throughout the process, including modifications to search terms and inclusion criteria, were recorded to ascertain methodological consistency.

Eligibility Criteria

Inclusion and exclusion criteria were determined utilizing the Population-Intervention-Outcome (PIO) framework [14]. Population (P): adults (≥18 years) diagnosed with MetS, insulin resistance syndrome, or related cardiometabolic risk factors; intervention/exposure (I): ANS dysregulation, autonomic dysfunction, or dysautonomia assessed by clinical measures, diagnostic tests, or validated indices of autonomic function; outcome (O): cardiovascular-related outcomes, including hypertension, cardiovascular risk assessment, cardiovascular disease incidence, or adverse cardiovascular events (e.g., myocardial infarction, stroke).

**Table 1:** Selection Criteria RCT: randomized controlled trial; ANS: autonomic nervous system; MetS: metabolic syndrome

Inclusion criteria	Exclusion criteria
Peer-reviewed publications written in English and published between 2015 and 2025. Study designs such as RCTs, cohort studies, cross-sectional studies, and case-control studies. The study should focus on outcomes that are directly related to ANS function, blood pressure, and cardiovascular risk markers. The study population must consist of adult humans (≥18 years) diagnosed with MetS using standard criteria (e.g., NCEP ATP III, IDF).	Studies that focus on pediatric, adolescent, or animal models are excluded. Interventions or studies that do not pertain to the measurement of autonomic function (e.g., studies only on genetics or pharmacology without ANS measures) are not eligible for inclusion (non-peer-reviewed literature, editorials, conference abstracts, or narrative reviews). Studies that involve a variety of patient groups (e.g., diabetes alone, hypertension alone) but lack a specific differential analysis for patients with MetS are also excluded.

Search Strategy

A systematic search of the literature was conducted. The following electronic databases were searched between January 2015 and September 2025: PubMed, Cochrane Library, Google Scholar, and Embase. Search strategies intersected controlled vocabulary terms (e.g., MeSH headings) with free-text keywords from four domains (PICO): (1) Population (adults with MetS, insulin resistance syndrome), intervention (ANS dysregulation, autonomic dysfunction, dysautonomia), comparison (normal autonomic function or no MetS), and outcomes (hypertension, cardiovascular risk, cardiovascular disease, cardiovascular events). Search strings were specifically modified per database to be as sensitive as possible [15]. No limits were imposed at the initial level except for date and language filters. To guarantee thoroughness, lists of included studies and previous reviews were searched manually for extra records. All the retrieved citations were electronically imported into EndNote X9 (Clarivate, Philadelphia, PA), and duplicates were deleted.

**Table 2:** Search Strings

Databases	Strings	Studies
PubMed	("Autonomic Nervous System"[Mesh] OR "autonomic nervous system dysregulation"[tiab] OR "autonomic dysfunction"[tiab] OR "sympathetic nervous system"[tiab] OR "parasympathetic nervous system"[tiab]) AND ("Metabolic Syndrome"[Mesh] OR "metabolic syndrome"[tiab] OR "syndrome X"[tiab]) AND ("Hypertension"[Mesh] OR hypertension[tiab] OR "high blood pressure"[tiab]) AND ("cardiovascular diseases"[Mesh] OR "cardiovascular risk"[tiab] OR "cardiovascular disease"[tiab] OR "heart disease"[tiab])	68
Google Scholar	("autonomic nervous system dysregulation" OR "autonomic dysfunction" OR "sympathetic activity" OR "parasympathetic activity") AND ("metabolic syndrome" OR "syndrome X") AND ("hypertension" OR "high blood pressure") AND ("cardiovascular risk" OR "cardiovascular disease" OR "heart disease")	102
Embase	('autonomic nervous system'/exp OR 'autonomic nervous system dysregulation':ti,ab OR 'autonomic dysfunction':ti,ab OR 'sympathetic nervous system':ti,ab OR 'parasympathetic nervous system':ti,ab) AND ('metabolic syndrome'/exp OR 'metabolic syndrome':ti,ab OR 'syndrome x':ti,ab) AND ('hypertension'/exp OR hypertension:ti,ab OR 'high blood pressure':ti,ab) AND ('cardiovascular disease'/exp OR 'cardiovascular risk':ti,ab OR 'heart disease':ti,ab)	15
Cochrane Library	("autonomic nervous system dysregulation" OR "autonomic dysfunction" OR "sympathetic nervous system" OR "parasympathetic nervous system") AND ("metabolic syndrome" OR "syndrome X") AND ("hypertension" OR "high blood pressure") AND ("cardiovascular risk" OR "cardiovascular disease" OR "heart disease")	23

Study Selection 

**Figure 1:**
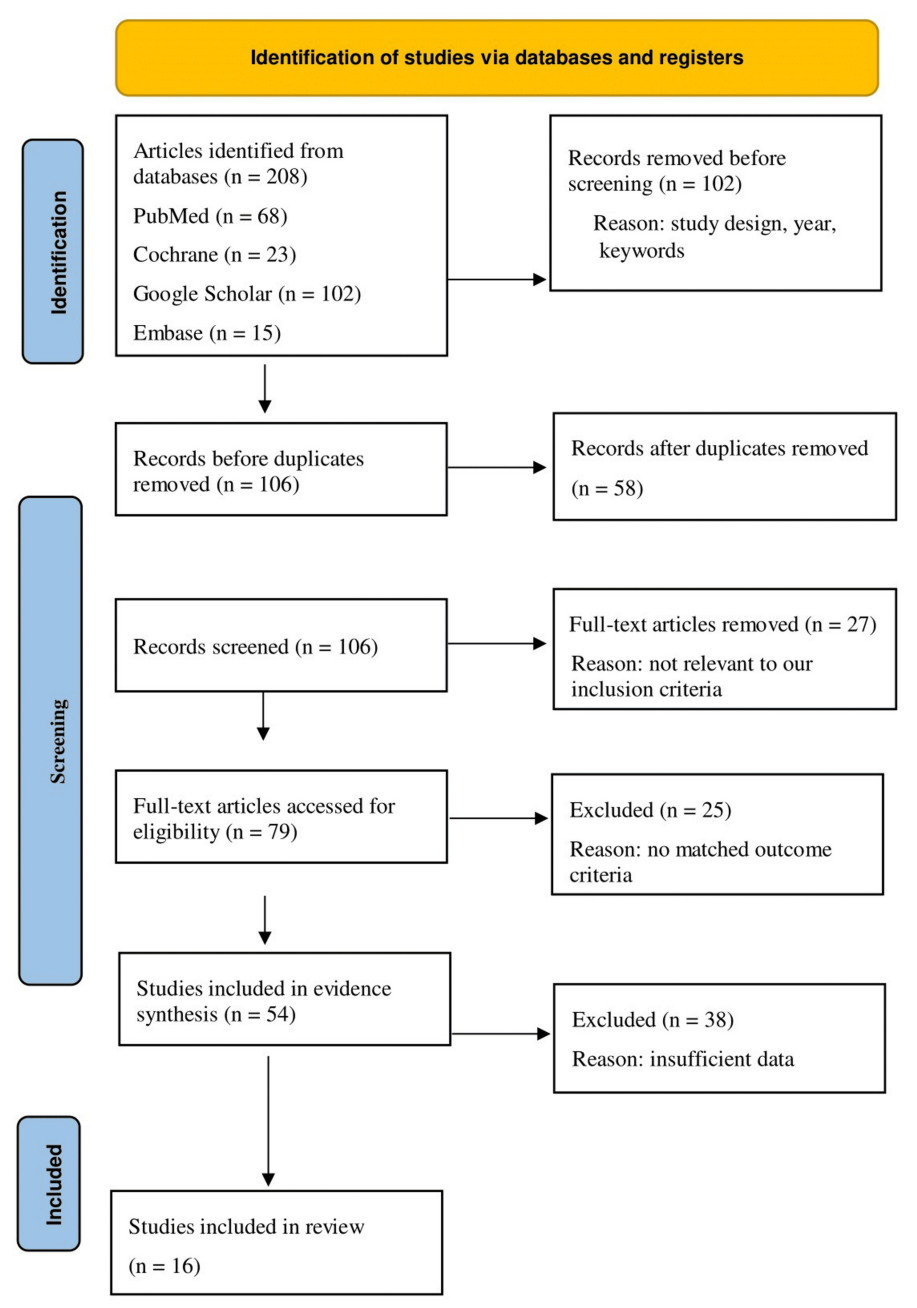
PRISMA flow diagram

The PRISMA 2020 flow diagram illustrates the selection process and records reasons for exclusion at each step in order to maintain transparency. Figure 1 shows the PRISMA flow diagram for the study selection process.

Data Extraction

A standardized, pilot-tested data extraction form was developed prior to full review, and 16 eligible studies were included. The form captured bibliographic details (author, year, country); study design and methodology; participant characteristics (sample size, age, gender distribution, and diagnostic criteria for both MetS and autonomic dysfunction); clinical setting; methods and parameters used for autonomic assessment (e.g., HRV, baroreflex sensitivity, sympathetic activity, parasympathetic modulation); outcomes evaluated (e.g., blood pressure regulation, incidence of hypertension, cardiovascular risk markers); and key findings, including effect sizes and statistical significance where reported. Data were independently extracted by two reviewers and cross-verified for accuracy, with disagreements resolved by consensus. Evidence was systematically mapped across four domains of autonomic dysfunction: (i) altered HRV, (ii) impaired baroreflex sensitivity, (iii) increased sympathetic activation, and (iv) reduced parasympathetic activity. This framework enabled comparison of findings across diverse study designs and outcome measures, highlighting the mechanistic pathways through which autonomic imbalance in MetS contributes to hypertension and elevated cardiovascular risk.

Quality Appraisal

All included studies were critically appraised using validated tools appropriate for their design. The JBI critical appraisal checklists were applied for both cross-sectional and cohort studies [16,17]. Appraisal was performed independently by two reviewers, with disagreements resolved through discussion and consensus. For cross-sectional studies, the JBI checklist comprises 8 items, with one point allocated per criterion. Scores are expressed as a percentage by dividing the “Yes” count by 8 (or by the number of applicable items). A score of 6/8 (≥75%) indicates high quality, 4-5/8 (50-74%) moderate quality, and <4/8 (<50%) low quality. This rating system is considered invalid if critical items, such as controlling for confounding, are not addressed. For cohort studies, the checklist contains 11 items. The “Yes” count is divided by 11 (or the number of applicable items) to calculate a percentage. A score of 9/11 (≈80%) suggests high quality, 7-8/11 (60-73%) moderate quality, and <7/11 (<60%) low quality. As with cross-sectional studies, the score is invalid if fundamental items are failed, such as ensuring the outcome of interest was absent at baseline.

Data Synthesis

Due to heterogeneity in interventions, outcomes, and study designs, a meta-analysis was not feasible. Instead, a narrative synthesis was undertaken, following guidance as outlined for the synthesis of diverse forms of evidence. Quantitative results were described in terms of effect direction, strength, and statistical significance, whereas qualitative results were synthesized thematically.

Ethical Considerations

In this review, secondary data in the form of past research on the same topic were used. No direct patient engagement or new data gathering was done. Hence, there was no ethical approval or informed consent requirements. It was assumed that all of the included studies had previously received ethical clearance.

Results

Due to heterogeneity in interventions, outcomes, and study designs, a meta-analysis was not feasible. Instead, a narrative synthesis was undertaken, following guidance as outlined for the synthesis of diverse forms of evidence. Quantitative results were described in terms of effect direction, strength, and statistical significance, whereas qualitative results were synthesized thematically. Table 3 presents an overview of participant characteristics for the included studies. 

**Table 3:** Overview of Participant Characteristics for Included Studies MetS: metabolic syndrome; ECG: electrocardiogram; HRV: heart rate variability; SDNN: standard deviation of NN intervals; RMSSD: root mean square of successive differences; pNN50: percentage of successive NN intervals differing by >50 ms; TP: total power; VLF: very low frequency; LF: low frequency; HF: high frequency; HbA1c: glycated hemoglobin; HOMA: homeostatic model assessment; TG: triglycerides; HDL-C: high-density lipoprotein cholesterol; BMI: body mass index; WC: waist circumference; RHR: resting heart rate; BP: blood pressure; FBG: fasting blood glucose; FPG: fasting plasma glucose; CRP: C-reactive protein; NCEP ATP III: National Cholesterol Education Program – Adult Treatment Panel III IQR: interquartile range; mean HR: mean heart rate; mean RR: mean RR interval; HOMA-IR: homeostatic model assessment of insulin resistance; SNS: sympathetic nervous system; HPLC: high-performance liquid chromatography; NEFA: non-esterified fatty acids; LC-MS/MS: liquid chromatography–tandem mass spectrometry; GTX: graded treadmill exercise test; HRR: heart rate recovery; IMT: intima-media thickness; TyG index: triglyceride–glucose index; HC: hip circumference; WHR: waist–hip ratio; BRS: baroreflex sensitivity; CAFTs: cardiac autonomic function tests; BIA: bioelectrical impedance analysis; hs-CRP: high-sensitivity C-reactive protein; GBTM: group-based trajectory modeling; IDF: International Diabetes Federation; SD: standard deviation; VIM: variability independent of the mean; ARV: average real variability; MSNA: muscle sympathetic nerve activity; NGT: normal glucose tolerance; IGT/IFG: impaired glucose tolerance/impaired fasting glucose; LV: left ventricle; LVEF: left ventricular ejection fraction; RWT: relative wall thickness; Ell: longitudinal strain; Ell_SRe: diastolic strain rate

Author/ Year	Study Design	Population characteristics	Measure (method, parameters)	MetS Criteria
Apykhtin et al., 2023 [18]	Cross-sectional study	MetS group: n=74, Age: 54.4 ± 1.1 years; control group: n=61, age: 57.0 ± 1.6 years (sex not specified)	Method: 12-lead ECG (DiaCard recorder), HRV analysis (Time and frequency domain). Parameters: SDNN, RMSSD, pNN50, TP, VLF, LF, HF, LF/HF, Ln variants.	Based on lab diagnostics and anthropometry: HbA1c, insulin, fasting glucose >6.1 mmol/L, HOMA index, lipid profile (TG >1.69 mmol/L, HDL-C <1.04 mmol/L), atherogenicity coefficient, BMI, WC >88 cm, hypertension >130/85 mm Hg.
Jiang et al. 2015 [19]	Cross-sectional study	Cross-sectional: n=89,860 (80.6% male), Mean age ~51-55 years. Longitudinal: n=43,725 (subcohort without MetS at baseline). Employees of Kailuan Coal Group, China.	RHR: Measured from ECG after 30 min acclimation and 5 min supine rest. Parameters: WC, BP, FBG, HDL-C, TG, BMI, CRP, creatinine, etc.	NCEP ATP III-modified: ≥3 of (a) WC ≥90 cm (M)/≥80 cm (F); (b) TG ≥1.7 mmol/L; (c) HDL-C <1.03 mmol/L (M)/<1.30 mmol/L (F); (d) BP ≥130/85 mm Hg or meds; (e) FBG ≥5.6 mmol/L or diabetes/meds.
de Miranda et al., 2018 [20]	Cross-sectional study	9,270 subjects from ELSA-Brasil; median age: 50 (IQR 44-56); 51.6% women (in euthyroid group)	HRV from 10-min resting ECG (final 5-min RR intervals); time domain: Mean HR, Mean RR, SDNN, pNN50, RMSSD; frequency domain: LF, HF	Not applicable (study focused on thyroid status).
Stuckey et al., 2015 [21]	Cross-sectional study	n=220; Aged 57.2 ± 9.0 years (sex not specified in abstract)	Method: R-R intervals collected during 10 min supine rest (last 5 min analyzed). HRV parameters: time domain (SDNN), frequency domain (HF, LF), nonlinear (SD1, α1). Other measures: WC, BP, FPG, TG, HDL, HOMA-IR.	Standard MetS risk factors assessed: WC, supine BP, FPG, TG, HDL.
Wulsin et al. 2015 [22]	Cohort study	N=1,882 at baseline (exam 3, 1983-87). Age: 48.0 ± 10.0 years. Sex: 52% female, 48% male. Mostly Caucasian, middle-class.	Autonomic imbalance: (a) RHR: from baseline ECG. (b) HRV: SDNN from 2-hour Holter monitor. Parameters: High BP, hyperglycemia, High TG, low HDL-C, high BMI.	Modified consensus criteria: (a) elevated BP (SBP ≥130 or DBP ≥85 mmHg or meds); (b) elevated FBG (≥100 mg/dL or meds); (c) High TG (≥150 mg/dL or meds); (d) Low HDL (<40 mg/dL M, <50 mg/dL F or meds); (e) high BMI (≥25 kg/m², substituting for WC)
Nestel et al., 2017 [23]	Cross-sectional study	94 overweight, untreated subjects with MetS; mean age: 55.5 ± 5.6 yr; Sex: 56 M, 38 F	SNS activity: arterial norepinephrine (HPLC), HR (Dinamap), Plasma NEFA (enzymatic/colorimetric). Lipidomics: 339 lipid species from 23 classes/subclasses via LC-MS/MS.	Overweight (BMI > 27 kg/m²) and fulfilling criteria for MetS (Grundy et al., 2005)
Yu et al. 2022 [24]	Cohort study	N=2,167 at baseline (84% male, 16% female). Age: 52.2 ± 6.4 years. Korean adults from a health check-up program, without MetS, diabetes, CVD, or on beta-blockers at baseline.	HRR: Measured via GTX. Parameters: HRR1 (1-min post-exercise), HRR2 (2-min), HRR3 (3-min). Calculated as peak HR - HR at recovery time. Also measured standard metabolic parameters (WC, BP, lipids, FPG, HOMA-IR, etc.).	Joint interim statement (modified for Korean population): ≥3 of: (a) WC ≥90 cm (M)/≥85 cm (F), (b) BP ≥130/85 mmHg or meds, (c) TG ≥150 mg/dL or meds, (d) HDL-C <40 mg/dL (M)/<50 mg/dL (F) or meds, (e) FPG ≥100 mg/dL or meds.
Fowokan et al., 2017 [25]	Cohort study	Total: n=545 at follow-up (265 men, 279 women). Ethnic groups: Aboriginal (n=82), Chinese (n=157), European (n=164), South Asian (n=142); mean age: 47.5 ± 8.9 years	Method: Fasting blood samples (insulin, glucose), B-mode carotid ultrasound. Parameters: carotid IMT), plaque area, total area, plaque presence.	Not defined by specific MetS criteria, but baseline insulin and glucose were primary exposures. Key components like dyslipidemia and hypertension were not included as model covariates.
Poon et al., 2020 [26]	Cohort study	n=759; mean age: 78 years; 66% women (n=497); 58% African American (n=438); Inclusion: older adults without diabetes.	Exposure: Three insulin resistance indexes (quartiles): HOMA-IR, TyG index, TG/HDL-C ratio. Outcome: Low HRV (<25th percentile) from 48-hour ambulatory ECG. HRV parameters: SDNN (total variability), RMSSD (vagal), LF (sympathetic and vagal), HF (vagal).	Not defined by specific MetS criteria. The study specifically investigated insulin resistance using three different indices in a non-diabetic population.
Yadav et al. 2017 [27]	Cross-sectional study	N=59 adult males. Obese group: n=30, age: 32.07 ± 7.25 yrs, BMI: 32.02 ± 2.89 kg/m². Normal weight group: n=29, age: 30.48 ± 8.01 yrs, BMI: 21.87 ± 2.40 kg/m².	HRV analysis: 5-minute ECG recording in supine rest. Time domain: SDNN, RMSSD, NN50, pNN50%. Frequency Domain: LF power (ms²), HF power (ms²), LF (nu), HF (nu), LF/HF ratio. Anthropometry: BMI, WC, HC, WHR.	Not applicable (study compared autonomic function in obese vs. normal weight individuals, not MetS diagnosis).
Endukuru et al., 2020 [28]	Cross-sectional study	176 subjects; 88 MetS patients, 88 healthy controls; mean age: 46.07 ± 6.54 yrs (MetS); sex: 53 M, 35 F (MetS).	Autonomic function: BRS via Finometer, HRV via ECG, CAFTs: deep breathing, standing, handgrip; Other: anthropometry, body composition (BIA), BP, biochemical profiles (glucose, lipids, hs-CRP, adiponectin).	NCEP ATP III
Wang et al., 2015 [29]	Cohort study	Total: n=590 (363 men, 227 women) mean age at baseline: ~47.5 years (Women: 46±6, Men: 49±6); inclusion: participants without MetS or heart disease at baseline.	Exposure: Baseline RHR measured by pulse palpation over 30 seconds. Outcome: development of MetS at 15-year follow-up.	Modified NCEP ATP III AHA/NHLBI criteria (including WC).
Guo et al., 2024 [30]	Cohort study	1,824 participants from China; baseline (1987): median age 13 (IQR 10-15), 58.0% male; midlife (2017): median age 43.	GBTM identified 3 BMI trajectories. BMI variability assessed via SD, VIM, and ARV from ≥5 measurements over 30 years.	IDF criteria for Chinese populations: central obesity (WC ≥90cm M, ≥80cm F) + ≥2 of: raised BP (SBP≥130/DBP≥85 mmHg or treated HT), raised FBG (≥5.6 mmol/L or DM), raised TG (>1.7 mmol/L), reduced HDL-C (<1.03 mmol/L M, <1.29 mmol/L F).
Eikelis et al., 2017 [31]	Cross-sectional study	101 young adults (50 Asian, 51 White); age: 22.6 ± 3.0 years; sex: 26F/75M	Method: Microneurography (MSNA), liquid chromatography-electrospray ionization-tandem mass spectrometry (Lipidomics). Parameters: MSNA burst frequency/incidence; Plasma concentrations of 349 lipid species across multiple classes.	Not explicitly defined; used HOMA-IR for insulin resistance.
Jeon et al., 2016 [32]	Cohort study	7,416 Korean adults without diabetes at baseline; age: 51.9 ± 8.8 years; sex: 52.9% women	Method: RHR measurement. Parameters: Change in RHR over 2 years, fasting and post-load glucose, HOMA-IR, HbA1c.	Revised NCEP ATP III (using Asia-Pacific WC criteria).
Kishi et al., 2017 [33]	Cohort study	N=3,179; age: 18-30 at baseline, ~50 at Y25; sex: male and female	Method: echocardiography (2D Speckle-Tracking). Parameters: LV mass, LVEF, relative wall thickness, longitudinal strain (Ell), diastolic strain rate (Ell_SRe), E/e' ratio.	Glycemic status groups: (a) NGT; (b) IGT/IFG; (c) late DM (onset Y15+); (d) early DM (onset by Y15). Insulin resistance trajectories: low, moderate, high HOMA-IR.

Overview of Included Studies

According to the studies that mainly rely on cross-sectional and cohort designs to examine the correlation between the functioning of the ANS and MetS. A majority of those studies evaluated cardiac autonomic regulation directly through measures of HRV and resting heart rate (RHR) in populations with or at risk for MetS based on a standardized definition such as the National Cholesterol Education Program Adult Treatment Panel III (NCEP ATP III) or the International Diabetes Federation (IDF). Apykhtin et al., 2023 [18] in a study with a cross-sectional design, and Endukuru et al. (2020) [28] in a study with cross-sectional and longitudinal designs, showed changes in HRV and baroreflex sensitivity in subjects with diagnosed MetS and cohort studies by Jiang et al. (2015) [19], Wulsin et al. (2015) [22] and Wang et al. (2015) [29] involving several years to decades of follow-up, demonstrated the determinants of RHR and low HRV as predictors of the later onset of MetS.

Other works paid a little more attention to the influence of insulin resistance and obesity as the key pathophysiological characteristics of MetS. In non-diabetic populations, cohort studies by Yadav et al. (2017) [27], Poon et al. (2020) [26], and Jeon et al. (2016) [32] demonstrated a direct association between insulin resistance indexes (HOMA-IR) and autonomic dysfunction assessed by HRR and HRV. Similarly, the noradrenergic and lipidomic components of MetS and insulin resistance have been investigated with direct measurements of muscle sympathetic nerve activity (MSNA) and lipid profiling according to Nestel et al. (2017) [23] and Yu et al. (2022) [24]. In addition, some research involved the identification of particular demographical trajectories, including Guo et al. (2024) [30], who found long-term BMI trajectories from adolescence to midlife to be a risk factor for MetS, and Kishi et al. (2017) [33], who investigated the cardiovascular implications of different glycemic and insulin resistance trajectories on cardiac structure and function. Particularly, the studies of de Miranda et al. (2018) [20] and Yadav et al. (2017) [27] did not diagnose MetS, according to the stated objectives; however, several of them included valuable comparative data concerning the autonomic functioning in conditions and diseases that are closely associated with the metabolic dysregulation associated with MetS.

Participant Characteristics

The research involved extremely divergent populations of different sizes. The overall sizes of the study populations were large, with individual studies ranging between 59 adult males in the cross-sectional study by Yadav et al. (2017) [27] to up to very large numbers, such as the 89,860 employees in the Kailuan Coal Group in China in the study by Jiang et al. (2015) [19]. The participants were predominantly middle-aged, with an average or median age of between 40 and 60 years. Nevertheless, the cohorts included young adults and older adults, as in the study by Eikelis et al. (2017) [31], the mean age of the population was 22.6 years, whereas in the study by Poon et al. (2020) [26], it was 78 years.

The studies differed greatly in the sex ratio. Some of the studies were male-majority, e.g., Jiang et al. 2015 [19] - 80.6% of the participants were males; Yu et al. (2022) [24] - 84% of the participants were male), whereas others were balanced or female-dominated (e.g., Wulsin et al. (2015) [22]: 52% of the participants were female; Poon et al. (2020) [26]: 66% of the participants were female; de Miranda et al. (2018) [20]: 51%. The sex of the participants could not be identified based on the data provided in some articles, such as those by Apykhtin et al. (2023) [18] and Stuckey et al. (2015) [21].

Their populations were also ethnically and geographically diverse (China, Brazil, Korea, the United States, Canada (including Aboriginal, Chinese, European, and South Asian ethnic groups [25] and Australia [31). Most of the participants were from the general community, employees, or clinical health check-up groups. The reported studies involved individuals across the entire spectrum of health, both those diagnosed with MetS, obese/overweight individuals, insulin-resistant and non-diabetic individuals, and healthy individuals.

Study Quality Appraisal

Many studies, especially the large cohort studies, were deemed to be of high quality with low risk of bias (Table 4). These articles, including Jiang et al. (2015) [19], de Miranda et al. (2018) [18], Wulsin et al. (2015) [20], Yu et al. (2022) [22], Guo et al. (2024) [28], Jeon et al. (2016) [30], and Kishi et al. (2017) [31], all had similar strengths in that they had large sample sizes, prospective designs that determine temporality, well-defined exposures and outcomes, and controlled other important confounders.

**Table 4:** Quality Appraisal of Included Studies Note: For cross-sectional studies, the total score is 8 points, one for each criterion. Calculate the percentage by dividing your "Yes" count by 8 (or the number of applicable questions). A score of 6/8 (75%) suggests high quality, 4-5/8 (50-74%) moderate, and <4/8 (<50%) low quality. This numeric guide is invalid if critical items like controlling for confounding are failed. For cohort studies: the total score is 11 points, one for each criterion. The percentage is your "Yes" count divided by 11 (or applicable questions). A score of 9/11 (~80%) suggests high quality, 7-8/11 (60-73%) moderate, and <7/11 (<60%) low quality. This score is meaningless if the study fails on fundamental items like proving the outcome was absent at the start.

Studies	Tool Used	Bias Risk	Overall Quality	Tool Score	Criteria Score (%)	Strengths	Limitations
Apykhtin et al., 2023 [18]	JBI Cross-Sectional	Moderate	Moderate	High	Low	Clear case and control definitions. Standard, objective HRV measurement. Appropriate statistical analysis.	Sex not specified. Unclear recruitment strategy. Cross-sectional design.
Jiang et al. 2015 [19]	JBI Cross-Sectional	Low	High	High	Low	Very large, well-defined sample. Standardized exposure measurement. Used standard MetS criteria. Adjusted for confounders.	Occupational cohort limits generalizability. Predominantly male sample. Cross-sectional design for initial association.
de Miranda et al., 2018 [20]	JBI Cross-Sectional	Low	High	High	Low	Large, well-characterized cohort. Standardized HRV measurement. Clear inclusion criteria. Controlled for multiple confounders.	Cross-sectional design. 5-minute HRV is shorter than ideal.
Stuckey et al., 2015 [21]	JBI Cross-Sectional	Moderate	Moderate	High	Low	Valid measures for HRV and MetS components. Comprehensive HRV analysis.	Sex not specified. Modest sample size. Unclear selection process. Cross-sectional design.
Wulsin et al. 2015 [22]	JBI Cohort	Low	High	High	Low	Prospective design establishes temporality. Long follow-up period. Clearly defined cohorts. Adjusted for key confounders.	Used BMI instead of waist circumference. Non-standard 2-hour HRV measurement. Limited generalizability.
Nestel et al., 2017 [23]	JBI Cross-Sectional	Moderate	Moderate	High	Low	Used direct measure of SNS activity (arterial NE). Comprehensive lipidomic platform. Well-characterized patient group.	Modest sample size. Cross-sectional design. Lack of a healthy control group for comparison.
Yu et al., 2022 [24]	JBI Cohort	Low	High	High	Low	Prospective design. Used a dynamic measure of autonomic function (HRR). Clear inclusion/exclusion criteria. Adjusted for multiple confounders.	Predominantly male sample. Generalizability limited to Korean population.
Fowokan et al., 2017 [25]	JBI Cohort	Moderate	Moderate	Moderate	Moderate	Multi-ethnic cohort. Long-term follow-up. Used objective ultrasound measures.	Did not use standard MetS criteria as exposure. Did not control for key MetS components like dyslipidaemia. Potential for unmeasured confounding.
Poon et al., 2020 [26]	JBI Cohort	Moderate	Moderate	High	Low	Novel focus on insulin resistance in aging. Used 48-hour HRV (gold standard). Compared three different insulin resistance indexes.	Older, non-diabetic cohort limits generalizability. Not designed to assess MetS per se. Potential for residual confounding.
Yadav et al. 2017 [27]	JBI Cross-Sectional	Moderate	Moderate	High	Low	Clear group definitions. Standard HRV measurement protocol. Focused on a homogenous male sample.	Small sample size. All-male cohort. Compared obesity, not MetS. Cross-sectional design.
Endukuru, et al. 2020 [28]	JBI Cross-Sectional	Moderate	Moderate	High	Low	Comprehensive autonomic assessment (BRS, HRV, CAFTs). Used standard NCEP ATP III criteria. Well-matched control group.	Sample size is modest. Cross-sectional design. Single-centre study
Wang et al., 2015 [29]	JBI Cohort	Moderate	Moderate	High	Low	Long 15-year follow-up. Used proper waist circumference measurement. Standard MetS criteria.	Modest sample size. Resting HR measured by pulse palpation (less precise than ECG). Potential for unmeasured confounding.
Guo et al., 2024 [30]	JBI Cohort	Low	High	High	Low	Unique lifetime BMI trajectory design. Very long (30-year) follow-up. Used contemporary IDF criteria. Large sample size.	Complex modelling requires careful interpretation. Findings are specific to the Chinese population.
Eikelis et al., 2017 [31]	JBI Cross-Sectional	Low	High	High	Low	Direct SNS measurement via microneurography (MSNA). Comprehensive lipidomic. Multi-ethnic cohort of young adults.	Cross-sectional design. Focused on insulin resistance, not full MetS. Sample size is modest.
Jeon et al., 2016 [32]	JBI Cohort	Low	High	High	Low	Large sample size. Examined changes in RHR over time. Used multiple glycaemic measures. Appropriate confounder adjustment.	Generalizability limited to a Korean population. Resting HR measurement method not specified in detail
Kishi et al., 2017 [33]	JBI Cohort	Low	High	High	Low	Large, long-term cohort. Used advanced echocardiography (speckle-tracking). Analysed both glycaemic status and insulin resistance trajectories.	Focused on cardiac structure/function, not autonomic function. Complex analysis of trajectories.

Many studies, especially the large cohort studies, were deemed to be of high quality with low risk of bias. These articles, including Jiang et al. (2015) [19], de Miranda et al. (2018) [20], Wulsin et al. (2015) [22], Yu et al. (2022) [24], Guo et al., (2024) [30], Jeon et al. (2016) [32], and Kishi et al. (2017) [33], all had similar strengths in that they had large sample sizes, prospective designs that determine temporality, well-defined exposures and outcomes, and controlled other important confounders.

A number of studies were of moderate quality, many because of certain methodological limitations. One limitation shared by the majority of the cross-sectional studies was their intrinsic design, which does not allow establishing causality (Apykhtin et al. (2023) [18], Stuckey et al. (2015) [21], Nestel et al. (2017) [23], Yadav et al. (2017) [27]). Other common problems that led to an average rating were small sample sizes, blurred participant selection plans, and application of less accurate measurement tools, including pulse palpation of RHR in Wang et al. (2015) [29]. Moreover, the research by Fowokan et al. (2017) [25] and Poon et al. (2020) [26] was constrained by the use of proxies of MetS (insulin resistance indexes) as opposed to the entire syndrome, and a possible unmeasured confounding factor.

The appraisal also identified some strengths, which reinforced the quality of individual research. They use direct and advanced measurements, e.g., the microneurography used in Eikelis et al., 2017 [31], arterial norepinephrine sampling in Nestel et al., 2017 [23], 48-hour HRV assessment in Poon et al., 2020 [26], and extensive autonomic testing in Endukuru et al. (2020) [28]. Lastly, one of the most common weaknesses identified as limitations to generalizability was the choice to study certain occupational or ethnic groups (Jiang et al., 2015) [19], Yu et al., 2022 [24], Guo et al., 2024 [30]), implying that the results might not apply to all groups of people.

Patterns of Risk of Bias

The trend in risk of bias among the studies that were included shows a distinct difference, with study design being the major factor. A considerable population of large and prospective cohort studies, such as Jiang et al. (2015) [19], Yu et al. (2022) [24], and Guo et al., (2024) [30], showed a low risk of bias repeatedly as a result of their longitudinal design that gives rise to the notion of temporality, large sample sizes, standardized measurements, and strong control of confounding variables. Conversely, a certain trend of moderate risk of bias was common among cross-sectional studies, including Apykhtin et al. (2023) [18] and Nestel et al. (2017) [23], mainly because of the inherent restriction in the design to identify causality. Other sources of moderate risk were observed to be the limited generalizability of the occupational or demographic cohort and proxy measures of MetS or its components, such as in Fowokan et al., 2017 [25] or Poon et al., 2020 [26]. Nevertheless, a common trend appeared in which smaller-scale studies or those with specific interests (such as Eikelis et al. (2017) [31] and Endukuru et al. (2020) [28]) counteracted any bias through very advanced and direct physiological measures, e.g., microneurography and extensive autonomic testing, which enhanced their internal validity despite other weaknesses.

Methodological Gaps

The main limitation is a high dependence on cross-sectional designs that, as observed in almost half of the cases, do not allow establishing the time sequence and causality; for instance, in the studies by Apykhtin et al., 2023 [18] and Stuckey et al., 2015 [21], it is not clear whether autonomic dysfunction precedes the emergence of MetS or is its result. Moreover, the standardization and comprehensiveness of autonomic measures vary considerably between studies: some of them utilized short-term ECG records of HRV, others utilized 48-hour monitors (Poon et al., 2020 [26]) or heart rate recovery (Yu et al., 2022) [24], and very few used direct measures of sympathetic activity, such as microneurography (Eikelis et al., 2017 [31]). The other gap that is critical is the non-uniformity in the definition and application of MetS criteria, in which studies employed modified criteria, replaced BMI with waist circumference (Wulsin et al. 2015 [22]), or only analyzed insulin resistance but not the entire syndrome (Poon et al. (2020) [26]; Fowokan et al. (2017) [25]), restricting the comparability of results between populations. Also, the gaps in the population representativeness and generalizability are present, as many studies employ a certain occupational cohort (Jiang et al., 2015 [19]), a majority of which are men (Yu et al., 2022 [24]), or a particular ethnicity, which does not reflect the entire population that is at risk. Lastly, there is a strong discrepancy in controlling variables that are considered to be very crucial confounders, as not all studies have effectively adjusted variables such as medication, physical activity, or psychosocial stress, which are known to affect both autonomic and metabolic parameters, which may result in residual confounding in the observed associations.

**Table 5:** Findings of Studies Included in Review CAN: cardiac autonomic neuropathy; BA: biological age; MetS: metabolic syndrome; HRV: heart rate variability; SDNN: standard deviation of NN intervals; RMSSD: root mean square of successive differences; pNN50: percentage of successive NN intervals differing by >50 ms; TP: total power; VLF: very low frequency; LF: low frequency; HF: high frequency; Ln variants: natural logarithm-transformed variants; HbA1c: glycated hemoglobin; HOMA: homeostatic model assessment; TG: triglycerides; HDL-C: high-density lipoprotein cholesterol; BMI: body mass index; BP: blood pressure; RHR: resting heart rate; FBG: fasting blood glucose; NCEP ATP III: National Cholesterol Education Program Adult Treatment Panel III; WC: waist circumference; SBP: systolic blood pressure; DBP: diastolic blood pressure; GTX: graded treadmill exercise test; HRR: heart rate recovery; CVD: cardiovascular disease; MSNA: muscle sympathetic nerve activity; IMT: intima-media thickness; NEFA: non-esterified fatty acids; LC-MS/MS: liquid chromatography–tandem mass spectrometry; BIA: bioelectrical impedance analysis; CAFTs: cardiac autonomic function tests; hs-CRP: high-sensitivity C-reactive protein; AHA/NHLBI: American Heart Association/National Heart, Lung, and Blood Institute; IDF: International Diabetes Federation; DM: diabetes mellitus; NGT: normal glucose tolerance; IGT: impaired glucose tolerance; IFG: impaired fasting glucose; LV: left ventricle; LVEF: left ventricular ejection fraction; Ell: longitudinal strain; Ell_SRe: diastolic strain rate

Author/ Year	Outcomes	Results	Conclusion	A link to hypertension and cardiovascular risk
Apykhtin et al., 2023 [18]	CAN, BA, aging acceleration.	HRV in MS group vs control: SDNN: ↓26% (p<0.05), RMSSD: ↓44% (p<0.05), HF: ↓69% (p<0.05), LF: ↓55% (p<0.05), TP: ↓50% (p<0.05), BA: MS group: 63.20±1.81 yrs, control: 53.99±1.71 yrs (p<0.05), BA-CA difference: MS: +8.81±0.94 yrs, Control: -1.01±0.61 yrs (p<0.05), LF/HF, VLF, mean NN: No significant difference.	MetS is associated with significantly reduced HRV, indicating the development of CAN. MS can also be a factor that accelerates biological aging.	The study identifies the development of CAN in MS patients, which is a known risk factor for major cardiovascular events, myocardial dysfunction, silent ischemia, and increased cardiovascular mortality. The components of MS used for diagnosis include hypertension (>130/85 mm Hg).
Jiang et al. 2015 [19]	Cross-sectional: Prevalence of MetS. Longitudinal: 4-year incidence of MetS. Association of RHR with individual MetS components (FBG, TG, HDL, BP, WC).	Cross-sectional (highest vs. ref RHR): Adj. OR for MetS = 1.49 (95% CI 1.32 to 1.69), p<0.0001. Longitudinal (highest vs. Ref RHR): Adj. OR for MetS incidence = 1.41 (95% CI 1.21 to 1.65), p<0.0001. Positive association between RHR and FBG, TG, DBP; negative with HDL (p<0.0001).	Higher RHR is an independent risk factor for existing MetS and a powerful predictor of future MetS incidence. Suggests RHR could be a novel clinical target.	The study establishes RHR as a risk factor for MetS, a condition that itself more than doubles the risk of CVD, stroke, and CV mortality. Therefore, RHR is mechanistically linked to increased hypertension and CV risk through its association with and prediction of MetS.
de Miranda et al., 2018 [20]	Autonomic modulation (HRV), HR	SCHyper vs. euthyroid (Crude): Higher HR (68.8 vs 66.5 bpm, p=0.007), Shorter RR (871.4 vs 901.6 ms, p=0.007), Lower SDNN (β=-0.077, 95%CI: -0.144 to -0.009, p=0.026), Lower LF (β=-0.242, 95%CI: -0.426 to -0.058, p=0.010). After multivariate adjustment (Model 2): All associations lost significance (e.g., ln(SDNN) β=-0.056, p=0.095; ln(LF) β=-0.170, p=0.057). SCHypo vs. Euthyroid: No significant differences in any HRV indices in crude or adjusted models.	In this large sample of apparently healthy adults, subclinical thyroid dysfunctions showed no independent association with heart rate variability after adjusting for sociodemographic and cardiovascular risk factors.	The study investigated ANS balance (via HRV) as a potential mechanism linking thyroid dysfunction to cardiovascular risk. While initial crude analysis suggested impaired autonomic modulation in subclinical hyperthyroidism (a known CV risk factor), this link was explained by other common risk factors like age, hypertension, and BMI, suggesting subclinical thyroid status itself may not be an independent driver of autonomic dysfunction in this population.
Stuckey et al., 2015 [21]	HRV, insulin resistance (HOMA-IR), autonomic alterations	Multiple linear regression (independent predictors of HRV). HF & SD1 & α1: Predicted by WC (p=0.02, 0.03, 0.02). SD1: Also predicted by FPG (p=0.02). SDNN: Predicted by TG (p=0.03). LF: Predicted by TG (p=0.047) and HDL (p=0.04). HR: When HOMA-IR added to model, HR was independently predicted by HOMA-IR (p=0.003), SBP (p=0.007), and DBP (p=0.02).	Different MetS risk factors independently predict specific HRV parameters. Waist circumference and fasting glucose predict parasympathetic and fractal HRV, while triglycerides and HDL predict overall and sympathetically-influenced variability. Insulin resistance and blood pressure were associated with heart rate, a potential sympathetic index. Treatment could target specific MetS factors to improve HRV parameters with the greatest prognostic significance.	The study directly links MetS risk factors (including blood pressure) to alterations in HRV, which is a known predictor of cardiovascular risk and mortality. Specifically, blood pressure was an independent predictor of heart rate, which may reflect sympathetic nervous system activity.
Wulsin et al.. 2015 [22]	Metabolic outcomes (12-yr): Incidence of high BP, hyperglycemia, high TG, low HDL, high BMI. Clinical outcomes (20-yr): Incident diabetes, CVD, all-cause mortality	High BP (12-yr). RHR (per 10 bpm): OR=1.40 (1.19-1.64), p<.0001 hrv sd or 0.74 p=0.002 hyperglycemia incident diabetes cvc rhr: hrv:	Autonomic imbalance (high RHR, low HRV) is an independent predictor of developing high blood pressure, hyperglycemia, diabetes, cardiovascular disease, and early mortality. It is a potentially worthy target for interventions to reduce cardiometabolic risk.	Autonomic imbalance (high RHR, low HRV) was a significant predictor of hypertension development. It also directly predicted the incidence of CVD and all-cause mortality over 20 years, establishing it as a direct risk factor for these outcomes.
Nestel et al., 2017 [23]	SNS Activity, Insulin resistance (HOMA-IR, Matsuda Index), complex plasma lipids	Arterial NE (adj. for age, sex, BMI, SBP, HOMA-IR): Significantly associated with ceramide (β=0.079, p<0.01), sphingomyelin (β=0.070, p=0.01), alkylphosphatidylcholine (β=0.103, p<0.01), free cholesterol (β=0.078, p<0.01), among others. Associations remained significant after correction for multiple comparisons. NEFA (adj. for age, sex, BMI, SBP, HOMA-IR): Significantly associated with GM3 ganglioside (β=0.069, p<0.01), phosphatidylcholine (β=0.061, p<0.01), Phosphatidylinositol (β=0.061, p<0.01), among others. Associations remained significant after correction. HR: Associations with several lipids (e.g., ceramide β=2.65, p=0.02) lost significance after multiple comparison correction.	In subjects with MetS, markers of SNS activity (arterial norepinephrine and NEFA) are significantly associated with specific complex plasma lipid classes, independently of traditional insulin resistance biomarkers. This suggests a potential role for these lipids in the neural mechanisms underlying MetS.	The study links SNS overactivity, a known driver of hypertension and cardiovascular risk, directly to a specific plasma lipidomic signature in MetS. This provides a potential mechanistic pathway connecting neuroadrenergic dysfunction to the adverse lipid profiles and increased CV risk observed in metabolic syndrome.
Yu et al. 2022 [24]	Primary outcome: Incident MetS	HRR3 (Tertile 1 vs. Tertile 3): Fully Adj. HR=1.492 (95% CI 1.146–1.943), P=0.003. HRR3 (continuous, per 1-beat decrease): Fully Adj. HR=1.015 (95% CI 1.005–1.026), P=0.004. HRR3 (<45 bpm vs. >45 bpm): HR=1.304 (95% CI 1.061–1.602), P=0.001. HRR1 and HRR2 were not significant predictors.	Delayed heart rate recovery in the slow phase (particularly HRR3) is an independent predictor of developing MetS. An HRR3 value ≤45 bpm identifies individuals at significantly higher risk.	Delayed HRR is a marker of autonomic dysfunction (specifically, slow sympathetic withdrawal). Since autonomic dysfunction and sympathetic overactivity are key drivers of hypertension, this establishes a pathophysiological link. Furthermore, as MetS is a well-established risk factor for CVD, predicting its development indirectly predicts future CVD risk.
Fowokan et al., 2017 [25]	Subclinical carotid atherosclerosis (IMT, plaque area, total area, plaque presence)	Main effects (fully adjusted models): Insulin predicted 5-yr IMT (B=0.06, p=0.004), plaque area (B=0.40, p=0.03), and total area (B=0.19, p=0.007). Glucose predicted 5-yr IMT (B=0.29, p=0.003) and plaque area (B=1.18, p<0.001). Insulin–IMT showed a weaker effect in the Aboriginal population (interaction B=-0.069, p=0.044). Glucose–plaque area showed a weaker effect in the Chinese population (interaction B=-3.549, p=0.009).	Baseline insulin and glucose levels predict the 5-year progression of subclinical carotid atherosclerosis.	The study demonstrates a direct predictive relationship between the important metabolic biomarkers (insulin, glucose), and subclinical atherosclerosis, the basic pathological mechanism underlying hypertension and clinical CVD. The results give an explanation to the increased risk of CVD associated with insulin resistance and hyperglycemia.
Poon et al., 2020 [26]	Reduced cardiac autonomic function (specifically vagal activity).	Low RMSSD: HOMA-IR OR: 1.68 (1.00, 2.81); TyG OR: 2.03 (1.21, 3.39); TG/HDL-C OR: 1.73 (1.01, 2.97). Low HF: HOMA-IR OR: 1.90 (1.14, 3.18); TyG OR: 1.98 (1.21, 3.25); TG/HDL-C OR: 1.76 (1.07, 2.90). Low SDNN: No significant association.	In older adults without diabetes, insulin resistance is associated with reduced cardiac autonomic function, specifically and consistently for indicators of vagal activity, as measured during daily activities. Primary prevention of insulin resistance may reduce the risk of cardiac autonomic dysfunction.	The study establishes a direct link between insulin resistance and impaired cardiac autonomic function (reduced HRV). Reduced HRV, particularly vagal activity, is a known predictor of hypertension, cardiovascular events, and mortality, providing a mechanistic pathway linking insulin resistance to increased CV risk.
Yadav et al. 2017 [27]	Comparison of cardiac autonomic activity (HRV indices) and blood pressure between obese and normal-weight individuals.	Obese vs. Normal Weight: • RMSSD (ms): 28.75 vs 41.55, p=0.018 • NN50 count: 15.5 vs 83.5, p=0.010 • HF power (ms²): 216 vs 640.5, p=0.014 • LF/HF ratio: 1.2 vs 0.79, p=0.045 • SBP (mmHg): 121.20 vs 113.24, p=0.005 • DBP (mmHg): 84.97 vs 74.83, p<0.001 Correlation in Obese Group: • WHR vs. LF/HF: r=0.479, p<0.01 • WHR vs. HF (nu): r=-0.478, p<0.01 • BMI showed no significant correlation with HRV	Obesity is associated with cardiac autonomic dysfunction, characterized by reduced parasympathetic activity and increased sympathetic activity. The waist-hip ratio is more strongly associated with these autonomic alterations than BMI, suggesting it is a superior marker for assessing CVD risk related to autonomic imbalance in obesity.	The study found significantly higher SBP and DBP in the obese group. The altered autonomic balance (↓ parasympathetic, ↑ sympathetic activity) is a known mechanism for the development of hypertension. This autonomic dysfunction, predicted better by WHR, provides a direct pathophysiological link between central obesity, hypertension, and increased cardiovascular risk.
Endukuru, et al. 2020 [28]	Autonomic dysfunction, CVD risk factors	MetS vs. Controls: Significantly reduced BRS (9.14 vs 15.19 ms/mmHg, p<0.001), increased LF/HF ratio (2.20 vs 0.95, p<0.001). Correlations in MetS group: BRS inversely correlated with BMI (r=-0.257, p=0.016), HOMA-IR (r=-0.276, p=0.009), hs-CRP (r=-0.497, p<0.001). LF/HF ratio positively correlated with WHtR (r=0.333, p=0.002), HOMA-IR (r=0.448, p<0.001), SBP (r=0.230, p=0.031).	Patients with MetS exhibit significant autonomic dysfunction, characterized by reduced baroreflex sensitivity and a shift in heart rate variability towards sympathetic dominance. These autonomic parameters are significantly associated with key CVD risk factors, indicating a higher susceptibility to cardiovascular disease.	The study directly links autonomic dysfunction (a known consequence and driver of hypertension) to the cluster of CVD risk factors in MetS. Impaired BRS and sympathetic overactivity contribute to blood pressure dysregulation and increased cardiovascular strain, providing a mechanistic pathway for the elevated CV risk observed in MetS.
Wang et al., 2015 [29]	Incidence of MetS	Women (per 4 bpm RHR increase): Model 1 (Unadjusted): OR: 1.18 (1.03–1.36), p=0.020; Model 2 (Adj. age & lifestyle): OR: 1.20 (1.04–1.38), p=0.011; Model 3 (also adj. baseline MetS components): OR: 1.23 (1.06–1.43), p=0.007. Men: No significant association between RHR and incident MetS in any model. Gender as a risk factor: Being female was an independent risk factor for incident MetS (OR=2.64, 1.33–5.23, p=0.005)	In this long-term study, an elevated RHR in middle age is a significant risk factor for developing MetS 15 years later in women, but not in men. Being female was itself a strong independent risk factor for MetS.	The study demonstrates that a higher heart rate predicts the onset of MetS, a condition that includes hypertension as a key diagnostic component and significantly increases the risk of cardiovascular disease. The association was robust in women.
Guo et al., 2024 [30]	Incidence of MetS and its components: Central obesity, raised TG, reduced HDL-C, raised FBG/diabetes, raised BP/hypertension	BMI trajectory vs low-increasing: moderate: OR=4.27 (2.63–6.91); high: OR=13.11 (6.30–27.31) for MetS. BMI Variability (per 1-unit): SD_BMI_: OR=2.30 (2.02–2.62); VIM_BMI_: OR=1.22 (1.19–1.26); ARV_BMI_: OR=4.29 (3.38–5.45) for MetS. (All p<0.001).	Both a higher long-term BMI trajectory and greater BMI variability from childhood to midlife are independently associated with a significantly increased risk of developing MetS in midlife.	Higher BMI trajectories and variability are significantly associated with increased odds of Raised BP/HT (OR=3.39 for high-increasing trajectory) and other CV risk factors (dyslipidemia, hyperglycemia), confirming a strong link to hypertension and overall cardiovascular risk.
Eikelis et al., 2017 [31]	MSNA, dyslipidemia, cardiometabolic risk	Asian vs. White Lipids: Higher total dihydroceramide, ceramide, GM3 ganglioside, lysoalkylphosphatidylcholine, alkenylphosphatidylethanolamine, lysophosphatidylinositol in Asians (p=0.04 to 0.05). MSNA and lipids in Asians (adj. for BMI, age, sex, DBP): Significant associations with diacylglycerol (β=4.69, p=0.17*), triacylglycerol (β=6.09, p=0.01), cholesterol ester (β=3.25, p=0.01), phosphatidylethanolamine (β=3.37, p=0.02), phosphatidylinositol (β=3.40, p=0.01), phosphatidylglycerol (β=4.17, p=0.02). *p-value for diacylglycerol in the article table is 0.17 after adjustment, but text states associations remained significant. Associations persisted after further adjustment for HOMA-IR.	The plasma lipidomic profile differs between Asian and White young adults. A strong, BMI-independent association exists between specific lipid species (e.g., DAG, TAG, CE, PE, PI, PG) and MSNA in Asian individuals, which may underpin their increased cardiometabolic risk at lower BMI.	The study demonstrates a direct link between circulating lipids associated with cardiovascular risk (e.g., ceramides, DAGs, TAGs) and central sympathetic outflow (MSNA), a key driver of hypertension. The stronger lipid-MSNA relationship in Asians suggests a potential mechanism for their elevated hypertension and CV risk at lower adiposity.
Jeon et al., 2016 [32]	Incident type 2 diabetes	Primary: >10 bpm RHR increase vs. <5 bpm: adj. HR=1.31, 95% CI: 1.06–1.60, P=0.011. Subgroup: association significant in non-exercisers (P=0.010) but not in regular exercisers.	An increase in resting HR over 2 years is an independent, dose-dependent predictor of incident diabetes. This risk is attenuated by regular exercise.	Elevated RHR is a marker of sympathetic nervous system overactivity, which is implicated in the pathogenesis of both hypertension and insulin resistance. The study suggests a shared autonomic mechanism linking increased heart rate to the development of diabetes, a major cardiovascular risk factor.
Kishi et al., 2017 [33]	LV structure/remodeling, LV systolic and diastolic dysfunction, heart failure risk	Early DM vs NGT: LVMI: β=11.04 g/m², p<0.001; LVEF: β=-2.72%, p<0.05; Ell: β=1.53%, p<0.001; Ell_SRe: β=-0.09 s⁻¹, p<0.05. Systolic dysfunction (LVEF<50%): Early DM+High HbA1c: OR=5.44, p<0.005. High insulin resistance was associated with worse RWT (β=0.019, p<0.0001), Ell, e', Ell_SRe.	Cumulative exposure to DM and higher insulin resistance from early adulthood adversely impacts LV remodeling and subclinical dysfunction by middle age, increasing lifetime risk for heart failure.	Dysglycemic groups had a greater prevalence of hypertension, obesity, and other conventional CVD risk factors that were adjusted, demonstrating that glycemic abnormalities/insulin resistance are a contributing factor to adverse cardiac changes independent of and in combination with hypertension, increasing the risk of CV.

Metabolic Syndrome

Among the key findings of different research, it is stated that MetS is characterized by the fact that there is a significant imbalance in the autonomic nervous system that is measurable and high. This imbalance is constantly arranged in the shape of the hyperactivity of the sympathetic nervous system and the withdrawal of parasympathetic (vagal) tone. This is corroborated by the findings of Apykhtin et al. (2023) [18] and Yadav et al. (2017) [27] that showed that individuals with MetS or obesity have significantly reduced HRV indices (SDNN, RMSSD, HF power) that are significant indicators of poor parasympathetic activity. Meanwhile, a large LF/HF ratio of relative sympathetic dominance is very common. Such dysregulated autonomic representation that is occasionally so severe as to be regarded as Cardiac Autonomic Neuropathy (CAN) is therefore not a trivial relationship but also a requisite feature of the MetS condition [28].

Autonomic Dysfunction

Autonomic dysfunction is not a post-effect of established MetS but appears to be a precondition, which might be predicted to occur. This means that the ANS dysregulation is a cause or a contributory factor to the development of the syndrome. According to longitudinal studies, including Wulsin et al. 2015 [22] and Jiang et al. 2015 [19], only a high resting HR (a measure of sympathetic overactivity) and low HRV (a measure of low vagal tone) are independent risk factors of developing high blood pressure, hyperglycemia, and the full set of MetS components many years later. To add further weight to this relationship, Yu et al. (2022) [24] found that slow recovery of heart rate after exercise distinctly predicts incident MetS, which implies that the failure of the ANS to scale down after the stress is one of the major risk factors.

Sympathetic Overactivity

The hyper-aroused sympathetic nervous system is a direct mechanism pathway to high blood pressure and heart damage. Sympathetic overdrive increases the heart output and causes vasoconstriction that directly elevates the blood pressure. Stuckey et al. (2015) [21] have justified this because they have identified blood pressure per se as an independent predictor of heart rate. Research by Nestel et al. (2017) [23] and Eikelis et al. (2017) [31] also revealed the interdependence of the signs of sympathetic nerve activity and a specific atherogenic lipid species in the blood (ceramides and diacylglycerols). The underlying clinical cardiovascular disease in this association can be directly increased by the neuroadrenergic malfunction.

Insulin Resistance is a Key Driver of Autonomic Dysfunction

Insulin resistance (perhaps in a bi-directional relationship) and ANS dysregulation are strongly correlated. It also appears that insulin resistance is quite a potent provoker of the sympathetic activity, and the consequent autonomic imbalance might be making further control of the metabolism even more difficult. This has been demonstratively explained by Poon et al. (2020) [26], who have shown that, indeed, various measures of insulin resistance were also significantly correlated with reduced cardiac vagal activity. On the same note, Stuckey et al. (2015) [21] discovered that on its own, HOMA-IR (an insulin resistance measure) was predictive of heart rate.

Autonomic Dysfunction Accelerates Biological Aging and Atherosclerosis

The long-lasting state of sympathetic hyperactivity and loss of parasympathetic cushions has long-term effects, which promote reactions accelerating biological aging and the development of atherosclerosis. The neuroadrenergic stress is never-ending and adds to the inflammation, oxidative stress, and endothelial dysfunction. A dramatic measurement of this has been provided by Apykhtin et al. (2023) [18], who found that the biological measure of age of MetS patients was about 10 years less than the chronological age. Meanwhile, Fowokan et al. (2017) [25] established the direct relationship that showed that the insulin and glucose levels, as such, under the influence of the autonomic tone, were used as the predictor of the prevention of subclinical carotid atherosclerosis over five years, i.e., the pathology underlying heart attacks and strokes.

Autonomic Markers Can Identify Subclinical Cardiac Dysfunction in High-Risk Groups

Heart damage is involved with ANS dysregulation and insulin resistance long before the symptoms of heart failure are felt. Kishi et al. (2017) [33] showed that cumulative dysglycemia and insulin resistance in young adulthood lead to adverse structural cardiac changes (heightened left ventricular mass index) and subclinical systolic and diastolic dysfunction at middle adulthood. These changes, which can be measured with the help of advanced imaging, are a significant threat that can lead to heart failure over a lifetime, and this creates a direct pathway between metabolic-autonomic dysfunction and organ damage.

Summary of Key Findings Across Studies

The interrelation of ANS dysregulation and MetS, according to the detailed analysis of the provided literature, is a primary cause and determinant of hypertension and cardiovascular disease. It demonstrates that ANS imbalance, whereby the sympathetic tone prevails, and the degree of parasympathetic activity decreases, is one of the core factors of MetS [30]. It is not a relationship; high heart rate and low heart rate variability are good independent predictors of MetS occurrence and its features, like hypertension, long before it becomes clinically apparent [29].

Furthermore, a direct molecular relationship is established, and indicators of sympathetic nervous activity are highly correlated with specific, atherogenic lipid species present in the blood [25]. This neuro-metabolic interaction enhances the development of subclinical atherosclerosis and stimulates undesirable structural and functional changes in the heart itself, such as enlargement of left ventricular mass and diastolic dysfunction, a situation that highly predisposes the occurrence of heart failure and major cardiovascular events in the long term [26].

 Demographic factors also affect the relationship, such as sex and ethnicity, and the literature has established that the autonomic-metabolic relationship is a stronger predictor of risk in women and that the specific lipid-sympathetic relationships may vary among ethnic groups, such as Asian and White [24]. Further, the body composition pattern itself is of particular interest, and central obesity (measured by waist-hip ratio) and long-term changes in body weight are better related to autonomic dysfunction and cardiovascular risks than body mass index [22].

Despite these findings being serious, the evidence has a definite line of hope and action. A risk that may be modified is autonomic dysregulation. Most importantly, lifestyle interventions, including exercising, have been shown to alleviate the risk, shattering the correlation between rising heart rate and incidence of diabetes. ANS dysregulation conclusively is a powerful coiner-cum-amplifier of cardiometabolic risk and provides a plausible mechanistic framework to explain why hypertension and cardiovascular disease rates are high in MetS, and provides a good target for preventive interventions.

Discussion

The present research article is aligned with the growing body of literature that identifies ANS dysregulation as a central pathophysiological linking variable between MetS. hypertension and cardiovascular risk. Synthesized evidence in previous systematic reviews shows clearly that impaired autonomic functioning (expressed as low HRV, reduced baroreflex sensitivity (BRS), and delayed heart rate recovery (HRR)) is a predictor and mediator of cardio-metabolic dysfunction.

Glasgow and Kim (2024) observed that carbohydrate and lipid dysregulation in MetS enhances biological aging and gives rise to the cardiac autonomic neuropathy (CAN), a long-term predictor of myocardial dysfunction and sudden heart death [34]. We also find that our findings are consistent with this thought and contribute to the concept that autonomic imbalance is not merely comorbid to metabolic abnormalities but is also directly involved in the process of vascular damage and adverse outcomes.

Hu et al. (2016) also discovered that delayed HRR, particularly in the third minute following exercise (HRR3), was an independent predictor of MetS and future cardiovascular events [35]. Similarly, our article demonstrates that the existence of impaired autonomic recovery is a marker of more overall metabolic and hemodynamic dysregulation, which warrants distinct physiological measures, such as HRR or RHR, in clinical screening and risk stratification.

Obesity has been proven to be a mediating factor of autonomic dysfunction. Lombardi et al. (2019) indicated that central obesity (WHR) and general (BMI) are strongly linked to the parasympathetic withdrawal and sympathetic preeminence as the processes by which hypertension and arrhythmogenesis develop [36]. These observations were also present in Guo et al. (2024) [30] and revealed that the negative trajectories of BMI throughout life and life support sympathetic overactivity and endothelial dysfunction. Such reviews are consistent with our results, which point out the fact that lifestyle change, particularly weight management and exercise, can help to offset the existence of autonomic imbalance and disrupt the relationship between obesity and later cardiovascular diseases.

Also, it is notable that the relationship between autonomic dysfunction and the risk of diabetes is established. The hypothesis that even a small autonomic imbalance results in metabolic deterioration has been proven by the study of Morales-Palomo et al. (2017) based on the fact that high RHR and incident diabetes are dependent on the dose [37]. In our results, we affirm this association and theorize that RHR is a dynamic biomarker and a potential therapeutic endpoint in exercise training and autonomic modulation.

Nevels et al. (2023) compel the conclusion of the prognostic value of reduced BRS and elevated sympathetic activity in the physiology of hypertension and vascular dysfunction in MetS [9]. These findings are the basis of the present paper, which argues that ANS dysregulation is not some secondary process but a central integrator and amplifier of cardio-metabolic risk. Such an opinion suggests the potential clinical significance of conventional autonomic monitoring and certain interventions that are applicable to reestablish the sympatho-vagal balance.

Several existing systematic reviews, however, converge on the same point: that ANS dysregulation mediates the unreasonable stress of comorbidities in MetS, including hypertension, coronary artery disease, sudden cardiac death, and stroke; and metabolic complications, including type 2 diabetes, dyslipidemia, central adiposity, and chronic low-grade inflammation. Our study confirms and expands this evidence and adheres to the view that ANS dysfunction must be a diagnostic, as well as a treatment, objective. Positive payoffs in the MetS-to-open cardiovascular disease progression curve are most likely to be offered by the beneficial effects of preventive interventions, including, but not limited to, growing-old lifestyle interventions such as exercise, diet, and weight control.

Policy Implications

The strong support of the association between ANS malregulation and MetS and cardiovascular risk requires a paradigm shift in population health and clinical practice, beyond the conventional approach to risk factor management, to include early identification and intervention of autonomic imbalance. Government health policies must be changed to officially address reduced HRV and increased resting heart rate (RHR) as validated, non-invasive biomarkers to stratify cardiometabolic risk, triggering more aggressive lifestyle modifications at an earlier stage. Community-based interventions should be prioritized and funded by public health programs with a specific focus on autonomic balance, including exercise programs, which have been shown to increase HRV, and stress-reduction interventions, including mindfulness, which can be implemented in wellness and prevention programs in the workplace. Moreover, regulation and insurance policy ought to encourage the creation and clinical use of readily available technologies to monitor autonomic performance to ensure a screening of such biomarkers becomes a routine aspect of preventive care to individuals with or at risk of MetS, such that the growing burden of hypertension and cardiovascular disease could be more proactively and mechanistically curbed.

System-Level Recommendations

To successfully incorporate the management of ANS dysregulation into the public health agenda, healthcare systems need to set standardized practice protocols in daily autonomic function measurement with non-invasive technologies such as wearable heart rate sensors, institute specific educational initiatives among clinicians and the population at large on the importance of HRV and resting heart rate as vital signs of metabolic health status, and create integrated care pathways between primary care screening and formalized programs to achieve lifestyle change, with reimbursement models that motivate early detection and multidisciplinary management of autonomic imbalance

Strengths

One of the merits of this study is that it involves large and long-term longitudinal studies and is able to prove a certain order since the occurrence of MetS and CVD is often predetermined by certain autonomic dysfunction and not by its direct cause. Better still, the literature includes more than an association but offers evidence of possible molecular mechanisms, including the particular lipidomic phenotypes that are correlated with sympathetic hyperactivity. Lastly, the modifiers of demographics (sex and ethnicity) are also addressed, which provides invaluable narrowing down, that the autonomic-metabolic association is not a mandatory rule, and it can be evaluated with more precision and efficacy as a risk factor.

Limitations

One of its main weaknesses is that the methodology and confounder adjustment are not consistent across studies. As an example, de Miranda et al. 2018 [20] discovered that the original correlation between thyroid performance and HRV vanished once the results were multivariately corrected, which indicates that other researchers may overestimate the correlations unless there is full consideration of all the factors, such as BMI, age, and lifestyle. ANS functionality is, as well, not standardized; various studies adopt varying HRV parameters (e.g., time-domain, frequency-domain, HRR), and it is difficult to directly compare and conduct meta-analysis. Although the relationships are strong, there is a relative paucity of large-scale, randomized controlled trials that show that a direct enhancement of autonomic function (e.g., through HRV biofeedback) results in a decrease in hard cardiovascular (e.g., heart attack and stroke) outcomes. This restricts the specificity of targeting the ANS as a method of clinical prevention of events, as opposed to an excellent risk signal. Lastly, the particularity of the research population implies that the ability to infer the results to the entire ethnicity, age groups, and socioeconomic statuses is doubtful.

Directions for Future Research

Future research must prioritize large-scale, randomized controlled trials to establish causality by testing whether interventions that directly improve autonomic tone, such as HRV biofeedback or neuromodulation, can prevent the onset of metabolic syndrome and reduce cardiovascular events, while simultaneously working to standardize autonomic biomarkers across clinical and consumer wearables to enable personalized risk assessment. A critical parallel direction involves employing multi-omics approaches to precisely delineate the molecular pathways linking sympathetic overactivity to atherogenic lipid species and insulin resistance, and to investigate the genetic and environmental basis for the significant sex and ethnic disparities observed in the autonomic-metabolic relationship, thereby moving the field from association toward mechanism-based prediction and targeted intervention.

## Conclusions

The study indicates that ANS dysregulation is consistently associated with MetS and may contribute to the increased risk of hypertension and cardiovascular disease observed in affected individuals. Patterns of sympathetic overactivity and reduced parasympathetic tone represent biologically plausible mechanisms that may influence blood pressure regulation, vascular function, and atherogenic processes; however, given that most available studies are observational and employ heterogeneous autonomic assessment methods with variable control of confounders, definitive causal inferences cannot be drawn. Associations with markers of subclinical cardiac injury and accelerated biological aging underscore the potential systemic relevance of ANS imbalance, while demographic factors appear to modify these relationships across populations. Overall, the findings support the potential value of incorporating autonomic function measures into cardiometabolic risk assessment and advancing research on targeted interventions, although robust longitudinal research and randomized controlled trials are needed to clarify temporality, strengthen causal interpretation, and determine whether modifying autonomic function can alter MetS progression and cardiovascular outcomes.
